# Impact of tobacco control policies on adolescent smokeless tobacco and cigar use: a difference-in-differences approach

**DOI:** 10.1186/s12889-018-5063-z

**Published:** 2018-02-15

**Authors:** Summer Sherburne Hawkins, Nicoline Bach, Christopher F. Baum

**Affiliations:** 1 Boston College, School of Social Work, McGuinn Hall, 140 Commonwealth Avenue, Chestnut Hill, MA 02467 USA; 2 Department of Economics, Boston College, Maloney Hall, 140 Commonwealth Avenue, Chestnut Hill, MA 02467 USA; 3 Department of Macroeconomics, German Institute for Economic Research (DIW Berlin), Mohrenstraße 58, 10117 Berlin, Germany

**Keywords:** Adolescent, Cigarette smoking, Taxes, Smoke-free policy, Smokeless tobacco, Cigars

## Abstract

**Background:**

While increasing cigarette taxes has been a major policy driver to decrease smoking, taxes on other tobacco products have received less attention. Our aims were to evaluate the impact of chewing tobacco/cigar taxes, cigarette taxes, and smoke-free legislation on adolescent male and female use of smokeless tobacco and cigars.

**Methods:**

We analyzed data on 499,381 adolescents age 14-18 years from 36 US states in the Youth Risk Behavior Surveys (1999-2013) linked to state-level tobacco control policies. We conducted difference-in-differences regression models to assess whether changes in taxes and the enactment of smoke-free legislation were associated with smokeless tobacco use and, separately, cigar use. Models were stratified by adolescent sex.

**Results:**

We found that chewing tobacco taxes had no effect on smokeless tobacco use and cigar taxes had no effect on cigar use. In contrast, among males a 10% increase in cigarette taxes was associated with a 1.0 percentage point increase (0.0010, 95% CI 0.0003-0.0017) in smokeless tobacco use. A 10% increase in cigarette taxes was also associated with a 1.5 percentage point increase (0.0015, 95% CI 0.0006-0.0024) in cigar use among males and a 0.7 percentage point increase (0.0007, 95% CI 0.0001-0.0013) in cigar use among females. There was some evidence that smoke-free legislation was associated with an 1.1 percentage point increase (0.0105, 95% CI 0.0015-0.0194) in smokeless tobacco use among males only, but no effect of smoke-free legislation on cigar use for males or females.

**Conclusions:**

Higher state cigarette taxes are associated with adolescents’ use of cheaper, alternative tobacco products such as smokeless tobacco and cigars. Reducing tobacco use will require comprehensive tobacco control policies that are applied equally to and inclusive of all tobacco products.

**Electronic supplementary material:**

The online version of this article (10.1186/s12889-018-5063-z) contains supplementary material, which is available to authorized users.

## Background

In spite of the decreasing trend in cigarette use among US adolescents, other tobacco products are gaining in popularity [1–4]. Data from 2013 to 2014 suggest that 6-9% of adolescents use smokeless tobacco and 8-13% smoke cigars [1, 5, 6]. Recent studies have shown that more high school students are using at least two tobacco products than cigarettes alone [1, 7]. There are known sex differences in adolescent use of these products, with males five times as likely to use smokeless tobacco and twice as likely to use cigars as females [1, 6]. The use and frequency of all tobacco products also escalates across the teenage years [1, 2, 4–8]. Among high school students the prevalence of smokeless tobacco and cigar use is 3-4 fold higher than middle school students [1].

While increasing cigarette taxes has been a major policy driver to decrease smoking [2, 9], including adolescent smoking [10], taxes on other tobacco products have received less attention. We identified only 3 studies in the peer-reviewed literature that examined the effects of state excise taxes on adolescent smokeless tobacco use [11, 12] or cigar use [13] and all used data from 2001 or prior. Two econometric analyses found that higher smokeless tobacco taxes were associated with lower use and frequency of smokeless tobacco [11, 12]. However, both studies were only conducted with males and neither included smoke-free legislation. In a cross-sectional study, Ringel and colleagues reported that higher cigar taxes were associated with lower cigar use [13]. They also found no evidence for an association between cigar use and cigarette excise taxes or smoke-free legislation.

To address these limitations in the evidence base and help inform public policy, we used state-representative data from 1999 to 2013 to evaluate the impact of chewing tobacco/cigar taxes, cigarette taxes, and the enactment of smoke-free legislation on adolescent male and female use and frequency of smokeless tobacco and cigars.

## Methods

The Youth Risk Behavior Survey (YRBS) is a state-representative survey that is conducted biennially in the US to monitor the health risk behaviors among 9th through 12th grade students. The YRBS produces state-level representative samples of students who are enrolled in public and private high schools. Students’ participation is anonymous and voluntary and they complete a self-administered survey during the school day. States must obtain a minimum response rate of 60% for data to be included [6, 14]. Further documentation has been published on the YRBS methodology [6, 14].

We analyzed 8 YRBS waves of data on 552,621 adolescents from 1999 to 2013 for the 36 states that included questions on smokeless tobacco and cigar use (Table 1). The state taxes for these products were also based on a percentage rather than an ad valorem tax. We excluded adolescents with missing information on cigarette use (28,987), smokeless tobacco use (7645), cigar use (5488), race (11,413), strata (4237), sex (3315), or age (1583), or the adolescent was younger than 14-years-old (4011). The final analytic sample included 499,381 adolescents. Adolescents were more likely to have data missing if they smoked cigarettes, were males, or identified as Black, Hispanic, or other race/ethnicity (*p* < 0.01).

**Table 1:** Adolescent smokeless tobacco and cigar use and tobacco control policies by state (*N* = 499,381)

	Years	*N*	%^a^	Mean %^a^Use smokeless tobacco	Mean %^a^Use cigars	12/2013 Cigarette tax (%)	2013 Chewing tobacco tax (%)	2013 Cigar tax (%)	100% Smoke-free restaurants
Alaska	03,07-13	6001	0.5	8.9	7.6	36.3	75%	75%	
Arkansas	99,01,05-13	9894	1.9	11.0	14.8	39.4	68%	68%	
Delaware	03-13	14,282	0.5	5.0	11.1	44.7	15%	15%	11/27/2002^c^
Florida	03-09	17,362	6.4	5.1	11.7	42.3	85%	0%	7/1/2003^c^
Georgia	03-13	10,832	6.2	6.6	12.1	30.6	10%	23%	
Idaho	03-13	9721	1.1	8.2	10.6	33.0	40%	40%	7/1/2004
Illinois	07-13	11,018	8.5	5.8	11.9	43.6	36%	36%	1/1/2008^c^
Indiana	03-11	9171	4.4	8.3	14.7	38.3	24%	24%	7/1/2012^c^
Iowa	05,07,11	4182	2.3	8.4	12.6	41.2	50%	50%	7/1/2008^c^
Kansas	05-13	8632	2.1	8.0	11.2	34.9	10%	10%	7/1/2010^c^
Kentucky	03-11	10,879	2.1	13.6	14.9	33.9	-^b^	15%	
Louisiana	07-13	4001	2.3	7.8	10.5	29.4	20%	20%	1/1/2007^c^
Maine	01-07	5259	0.6	4.8	11.4	47.1	-^b^	20%	1/1/2004^c^
Maryland	05-13	53,968	3.7	4.0	10.0	47.3	30%	15%	2/1/2008^c^
Massachusetts	99-09,13	22,713	3.6	4.6	12.5	46.9	210%	40%	7/5/2004^c^
Michigan	99-13	25,946	6.9	6.7	12.4	47.1	32%	32%	5/1/2010^c^
Mississippi	99-03,07-13	10,799	1.9	7.7	14.7	34.5	15%	15%	
Missouri	99-09,13	10,913	4.0	7.9	14.1	27.8	10%	10%	
Montana	99-13	24,173	0.7	13.5	15.2	44.2	50%	50%	10/1/2005^c^
Nebraska	03,05,11,13	11,322	1.3	7.2	10.6	32.6	20%	20%	6/1/2009^c^
Nevada	99,13	3566	0.4	6.2	11.8	34.9	30%	30%	12/8/2006^c^
New Hampshire	03-13	8432	1.0	6.5	15.1	45.8	65.03%	65.03%	9/17/2007
New Jersey	01	1953	0.9	5.8	14.2	50.7	30%	30%	4/15/2006^c^
New Mexico	05-13	22,104	1.4	8.3	15.2	43.3	25%	25%	6/15/2007
New York	03-09,13	51,527	9.7	4.4	9.0	53.8	75%	75%	7/24/2003^c^
Ohio	99,03,11,13	5727	5.8	9.1	14.7	40.9	17%	17%	12/7/2006^c^
Rhode Island	01-13	15,806	0.7	4.1	10.2	54.8	80%	80%	3/1/2005^c^
South Carolina	99,05,09-13	9159	2.3	8.4	14.9	32.6	5%	5%	
South Dakota	99-03	4670	0.2	14.8	13.7	43.6	35%	35%	11/10/2010^c^
Tennessee	03-13	11,520	4.1	12.0	15.0	33.7	6.6%	6.6%	
Utah	99-13	12,142	2.2	2.8	4.5	43.4	86%	86%	1/1/1995^c^
Vermont	99-03,09	28,207	0.2	6.5	12.6	48.4	92%	-^b^	9/1/2005^c^
Virginia	11,13	7592	5.4	7.0	10.1	28.0	10%	10%	
West Virginia	99,03-13	10,649	1.2	13.8	13.4	32.8	7%	7%	
Wisconsin	99,01,05-13	14,657	3.5	8.3	14.8	48.8	71%	71%	7/5/2010^c^
Wyoming	99-03,11,13	10,602	0.3	14.8	14.9	32.9	20%	20%	

^a^ Weighted

^b^ Ad valorem tax

^c^ Also had legislation for smoke-free workplaces

### Outcome measures

Adolescents answered three questions about their tobacco product use. Adolescents were asked, “During the past 30 days, on how many days did you use chewing tobacco, snuff, or dip, such as Redman, Levi Garrett, Beechnut, Skoal, Skoal Bandits, or Copenhagen?”, with 7 responses (in days): 0, 1 or 2, 3 to 5, 6 to 9, 10 to 19, 20 to 29, 30. We defined current smokeless tobacco use as a dichotomous measure if adolescents responded 0 days (no) versus 1-30 days (yes). We also defined current smokeless tobacco use as a categorical variable based on frequency of responses indicating frequency: 0, 1-5, 6-29, 30 days per month.

Separately, adolescents were asked, “During the past 30 days, on how many days did you smoke cigars, cigarillos, or little cigars?” and “During the past 30 days, on how many days did you smoke cigarettes?”, with 7 responses: 0 days to all 30 days. For each tobacco product we constructed a dichotomous measure and a variable indicating frequency.

Students self-reported their age (14, 15, 16, 17, 18 years) and sex (male, female) and we constructed race/ethnicity (white, Black, Hispanic, Other). No additional socio-demographic information was collected consistently over survey years.

### State tobacco control policies

We linked tobacco control policies with each adolescent based on the state-year the survey was completed. We used the state tax on chewing tobacco for use of chewing tobacco, snuff or dip (referred to as smokeless tobacco) and the tax on cigars for use of cigars, little cigars, and cigarillos (referred to as cigars). The taxes from the fourth quarter of each study year were obtained from the State Tobacco Activities Tracking & Evaluation (STATE) System [15], as this was the only measure available from 1999 to 2005. Taxes for both products were based on a percentage of the retail sales price, manufacturer’s price/sales, or wholesale price/sales and states were consistent on the type of tax across the study period. While each measure may quantify different aspects of the tax, it was not possible to standardize percentages across the three measures. 29/36 states had the same percentage tax for both chewing tobacco and cigars.

To maintain consistency of units between tax measures, we included the annual cigarette tax (state and federal) as a percentage of the retail price from the *Tax Burden on Tobacco* [16]*.* We obtained information on 100% smoke-free legislation for workplaces and restaurants from the *American Nonsmokers’ Rights Foundation* [17]. Based on our prior methodology [10], we used 100% smoke-free restaurant legislation as a proxy for state smoke-free policies and adolescents were indicated as living in a state with smoke-free legislation if their state had restaurant legislation by March of the survey year [6, 14].

To control for differences in state tobacco control funding [18, 19], we included a measure of the annual appropriations/grants each state receives for tobacco control efforts [15]. As described previously [10], we calculated an annual per capita measure of state tobacco control funding by dividing the total funds for each state by the US census population estimates for all ages [15, 19].

### Statistical analysis

We first examined the socio-demographic characteristics of adolescents who currently used smokeless tobacco and estimated an adjusted logistic regression model to assess predictors of adolescent smokeless tobacco use by age, race/ethnicity, sex, and cigarette and cigar use. We included state and year fixed effects in all regression models to control for time-invariant state factors, such as tobacco production, and capture time trends in tobacco use [2, 20].

We estimated difference-in-differences models, a causal inference technique [21], to evaluate the impact of chewing tobacco and cigarette taxes and smoke-free legislation on adolescent smokeless tobacco use and, separately, frequency. To test the assumption of parallel trends, we plotted the prevalence of smokeless tobacco and cigar use, separately, by each policy: smoke-free legislation (comparing states that implemented smoke-free legislation with those that did not), chewing tobacco or cigar taxes (comparing states that increased taxes versus those that did not), and cigarette taxes (comparing states median cigarette tax as a percentage of the price). Overall, Additional file 1: Figures S1 and S2 illustrate similar trends in use of smokeless tobacco and cigars over the study period. We examined correlations between taxes on cigarettes, chewing tobacco and cigars ranging from *r* = 0.54-0.57 and the variance inflation factors were 1.61-1.71; thus, rejecting concerns about multicollinearity.

Since adolescent males are more likely to use smokeless tobacco and cigars than females [1, 6], we stratified all models by adolescent sex. We estimated sex-stratified fixed-effects probit regression models to assess whether changes in chewing tobacco taxes (*Chewtax*), cigarette taxes (*Cigtax*), and smoke-free restaurant legislation (*Sfrest*) were associated with changes in adolescent smokeless tobacco use (*Currchew*) as a dichotomous outcome, controlling for tobacco control funding (*Tobspend*), current cigarette (*Currsmoke*) and cigar (*Currcigar*) use, socio-demographic characteristics (*Age, Race*), state (*State*), and year (*Year*):

$$ {\displaystyle \begin{array}{l}\Pr \left[ Currchew=1\right]={\beta}_0+{\beta}_1 Chewtax+{\beta}_2 Cigtax+{\beta}_3 Sfrest+{\beta}_4 Tobspend+\\ {}{\beta}_5 Currsmoke+{\beta}_6 Currcigar+{\sum}_{i=15}^{18}{\gamma}_i{Age}_i+{\sum}_{i=B,H,O}{\lambda}_i{Race}_i+{\sum}_j{\phi}_j{State}_j+\\ {}{\sum}_k{\delta}_k{Year}_k+\varepsilon \end{array}} $$

We calculated average marginal effects to determine the change in the probability of adolescent smokeless tobacco use with a 10% tax increase or the enactment of smoke-free legislation.

We then estimated ordered probit regression models to assess the impact of changes in state tobacco control policies on the number of days/month that males and females used smokeless tobacco (0, 1-5, 6-29, 30 days). We calculated average marginal effects for each smokeless tobacco outcome category, which sum to zero.

This series of analyses was repeated for adolescent cigar use. Cigar tax was substituted for chewing tobacco tax and models were adjusted for both cigarette and smokeless tobacco use. Since the majority of states had the same percentage tax for both chewing tobacco and cigars, models only included the relevant tax rate. As a robustness check we only examined those states with tax changes for chewing tobacco (18 states) or cigars (16 states) from 1999 to 2013.

We used Stata statistical software, version 14.0 (StataCorp, College Station, TX), for all analyses. Based on YRBS documentation, we used survey (svy) commands to account for the complex sample design and obtain linearized standard errors [14]. We used the ‘subpop’ command for sex-stratified analyses. We included survey weights in all analyses to provide state-representative estimates [14]. Survey weights were calculated from adolescents’ race/ethnicity, gender, and school grade to adjust for nonresponse and oversampling of non-white adolescents [14].

## Results

Over the study period, mean smokeless tobacco use ranged from 2.8% in Utah to 14.8% in South Dakota and Wyoming and cigar use ranged from 4.5% in UT to 15.2% in Montana and New Mexico (Table 1). By the 4th quarter of 2013, the mean chewing tobacco tax was 42.8% of the price and the mean cigar tax was 32.6% of the price. By December 2013, the mean cigarette tax was 40.1% of the price and 25/36 states had 100% smoke-free restaurant legislation.

Males were 6.91 times more likely to use smokeless tobacco and 2.30 times more likely to use cigars than females (Table 2). Tobacco use also increased with age, as the odds of smokeless tobacco and cigar use nearly doubled from ages 14 to 18 years. While Black (adjusted OR = 0.22) and Hispanic (adjusted OR = 0.56) adolescents were less likely to use smokeless tobacco than white adolescents, Black adolescents (adjusted OR = 1.46) were more likely to use cigars. After controlling for socio-demographics and state fixed effects, there was an increasing trend in smokeless tobacco use and no change in cigar use over the study period. There was also strong evidence for the use of all three tobacco products, as the use of one product increased the odds of using the other two. Similar patterns were seen according to the number of days/month that adolescents used smokeless tobacco and cigars (Additional file 1: Table S1). Overall, the majority of adolescents who used these products reported doing so for only 1-5 days/month.

**Table 2:** Adolescent socio-demographic characteristics and predictors of current smokeless tobacco and cigar use (*N* = 499,381)

			Smokeless tobacco		Cigars	
	*N*	%^a^	Mean %^a^ Use	Adjusted OR^b^(95% CI)	Mean %^a^ Use	Adjusted OR^b^ (95% CI)
Age						
14	61,501	10.6	4.3	1	6.6	1
15	132,380	25.6	6.0	1.18 (1.08-1.30)	9.1	1.20 (1.11-1.30)
16	134,492	26.2	7.3	1.23 (1.12-1.35)	12.2	1.46 (1.36-1.58)
17	113,904	23.7	7.9	1.19 (1.08-1.31)	14.6	1.67 (1.55-1.80)
18	57,104	13.9	9.7	1.25 (1.13-1.39)	18.5	1.96 (1.81-2.13)
Race/ethnicity						
White	309,470	66.9	8.9	1	13.0	1
Black	69,774	18.3	2.0	0.22 (0.20-0.25)	10.2	1.46 (1.37-1.55)
Hispanic	69,890	9.5	4.9	0.56 (0.51-0.62)	11.1	1.02 (0.95-1.09)
Other	50,247	5.4	6.4	0.72 (0.67-0.78)	11.4	1.00 (0.94-1.08)
Sex						
Female	258,648	50.1	2.0	1	7.7	1
Male	240,733	49.9	12.3	6.91 (6.49-7.36)	16.8	2.30 (2.21-2.40)
Year						
1999	35,351	5.7	9.0	1	19.1	1
2001	32,793	4.6	7.2	1.16 (0.99-1.36)	13.6	0.76 (0.69-0.84)
2003	56,993	11.9	6.1	0.99 (0.83-1.17)	11.6	0.78 (0.71-0.86)
2005	60,235	13.7	6.7	1.18 (1.03-1.37)	12.5	0.92 (0.84-1.01)
2007	64,586	16.0	6.8	1.34 (1.16-1.55)	12.0	0.89 (0.81-0.98)
2009	73,039	15.6	7.5	1.58 (1.37-1.82)	12.6	0.97 (0.89-1.07)
2011	59,491	15.8	7.9	1.50 (1.29-1.75)	11.8	0.90 (0.82-0.99)
2013	116,893	16.7	6.8	1.60 (1.40-1.84)	10.3	0.95 (0.86-1.05)
Current cigarette use						
No	407,843	81.1	3.6	1	4.9	1
Yes	91,538	18.9	22.3	4.50 (4.23-4.77)	43.9	13.48 (12.91-14.07)
Current smokeless tobacco use						
No	464,132	92.9	–	–	9.4	1
Yes	35,249	7.1	–	–	49.2	3.42 (3.20-3.64)
Current cigar use						
No	441,355	87.8	4.1	1	–	–
Yes	58,026	12.3	28.6	3.41 (3.20-3.63)	–	–

*CI* confidence interval, *OR* odds ratio

^a^ Weighted

^b^ State fixed effects not shown

We found no evidence for an effect of chewing tobacco taxes on adolescent smokeless tobacco use (Table 3). However, among males, we found that a 10% increase in cigarette taxes was associated with a 1.0 percentage point increase in smokeless tobacco use (*p* = .008). Results were consistent when we examined frequency of use (Table 4). A 10% increase in cigarette taxes was associated with males being 1.0% less likely to use smokeless tobacco 0 days/month and comparatively 0.4% more likely for 1-5 days, 0.3% for 6-29 days, and 0.3% for 30 days (all *p* = .01). The enactment of smoke-free restaurant legislation was associated with an increase in the use of smokeless tobacco by 1.1 percentage points among males (*p* = 0.02). Regarding frequency of use, smoke-free legislation was associated with males being 1.0% less likely to use smokeless tobacco 0 days/month and comparatively 0.4% more likely for 1-5 days, 0.3% for 6-29 days, and 0.4% for 30 days (all *p* = .03). In contrast, we found no consistent evidence for the effect of cigarette excise taxes or the enactment of smoke-free legislation on female smokeless tobacco use or frequency.

**Table 3:** Marginal effects from sex-stratified fixed-effects probit regression models of the impact of state tobacco control policies on smokeless tobacco and cigar use (*N* = 499,381)

	Smokeless tobacco			Cigars		
	Mean %^a^ Use	Marginal effect of coefficient^b,c^ 95% CI	*p* Value	Mean %^a^ Use	Marginal effect of coefficient^b,d^ 95% CI	*p* Value
Males (*N* = 240,733)	12.3%			16.8%		
Tobacco tax (%)		0.0001 (−0.0001-0.0002)	.5		0.0001 (−0.0002-0.0004)	.4
Cigarette tax (%)		0.0010 (0.0003-0.0017)	.008		0.0015 (0.0006-0.0024)	.001
100% smoke-free restaurants (yes/no)		0.0105 (0.0015-0.0194)	.02		−0.0062 (− 0.0154-0.0030)	.2
Females (*N* = 258,648)	2.0%			7.7%		
Tobacco tax (%)		−0.0000 (− 0.0001-0.0000)	.3		0.0002 (− 0.0001-0.0004)	.1
Cigarette tax (%)		0.0003 (−0.0000-0.0006)	.09		0.0007 (0.0001-0.0013)	.02
100% smoke-free restaurants (yes/no)		0.0002 (−0.0042-0.0046)	0.9		−0.0002 (− 0.0070-0.0065)	.9

*CI* confidence interval

^a^ Weighted

^b^ Model includes adjustment for the following covariates: cigarette use, state tobacco control expenditure, age, race, state, and year

^c^ Model includes adjustment for cigar use

^d^ Model includes adjustment for smokeless tobacco use

**Table 4:** Marginal effects from sex-stratified fixed-effects ordered probit regression models of the impact of state tobacco control policies on the number of days/month used smokeless tobacco or cigars (*N* = 499,381)

	Number of days/month smokeless tobacco used				Number of days/month cigars used			
	Mean %^a^ Use	Marginal effect of coefficient^b,c^ 95% CI		*p* Value	Mean %^a^ Use	Marginal effect of coefficient^b,d^ 95% CI		*p* Value
Males (*N* = 240,733)								
0 days	87.7%							
1-5 days	5.3%				11.6%			
6-29 days	3.6%				3.6%			
30 days	3.4%				1.6%			
Tobacco tax (%)								
0 days		−0.0001 (−0.0003 − 0.0001)		.4		-0.0001 (−0.0004-0.0002)		.4
1-5 days		0.0000 (−0.0000-0.0001)		.4		0.0001 (−0.0001-0.0002)		.4
6-29 days		0.0000 (−0.0000-0.0001)		.4		0.0000 (−0.0000-0.0003)		.4
30 days		0.0000 (−0.0000-0.0001)		.4		0.0000 (−0.0000-0.0001)		.4
Cigarette tax (%)								
0 days		−0.0010 (− 0.0017--0.0002)		.01		−0.0015 (− 0.0023--0.0006)		<.001
1-5 days		0.0004 (0.0001-0.0006)		.01		0.0009 (0.0004-0.0014)		<.001
6-29 days		0.0003 (0.0001-0.0005)		.01		0.0003 (0.0002-0.0005)		<.001
30 days		0.0003 (0.0001-0.0006)		.01		0.0002 (0.0001-0.0003)		<.001
100% smoke-free restaurants (yes/no)								
0 days		−0.0099 (−0.0189-0.0009)		.03		0.0047 (−0.0041-0.0136)		.3
1-5 days		0.0036 (0.0003-0.0069)		.03		−0.0029 (− 0.0008-0.0025)		.3
6-29 days		0.0028 (0.0003-0.0053)		.03		−0.0011 (− 0.0003-0.0010)		.3
30 days		0.0035 (0.0003-0.0067)		.03		−0.0007 (− 0.0020-0.0006)		.3
Females (*N* = 258,648)								
0 days	98.0%				92.3%			
1-5 days	1.4%				5.9%			
6-29 days	0.3%				1.4%			
30 days	0.2%				0.5%			
Tobacco tax (%)								
0 days		0.0001 (−0.0000-0.0001)		.1		−0.0002 (−0.0004-0.0001)		.2
1-5 days		−0.0000 (− 0.0000 − 0.0000)		.1		0.0001 (− 0.0001-0.0003)		.2
6-29 days		−0.0000 (− 0.0000-0.0000)		.1		0.0000 (−0.0000 − 0.0003)		.2
30 days		-0.0000 (−0.0000-0.0000)		.1		0.0000 (−0.0000-0.0001)		.2
Cigarette tax (%)								
0 days		-0.0003 (−0.0006-0.0000)		.05		−0.0008 (− 0.0014--0.0002)		.007
1-5 days		0.0002 (−0.0000 − 0.0004)		.05		0.0005 (0.0001-0.0009)		.007
6-29 days		0.0001 (−0.0000-0.0001)		.05		0.0002 (0.0000-0.0003)		.007
30 days		0.0001 (−0.0000-0.0001)		.05		0.0001 (0.0000-0.0002)		.007
100% smoke-free restaurants (yes/no)								
0 days		-0.0004 (−0.0048-0.0039)		.8		0.0011 (−0.0054-0.0075)		.7
1-5 days		0.0003 (−0.0025-0.0030)		.8		−0.0007 (− 0.0048-0.0034)		.7
6-29 days		0.0001 (−0.0008-0.0009)		.8		−0.0002 (− 0.0017-0.0012)		.7
30 days		0.0001 (−0.0006-0.0008)		.8		−0.0001 (− 0.0010-0.0007)		.7

*CI* confidence interval

^a^ Weighted

^b^ Model includes adjustment for the following covariates: cigarette use, state tobacco control expenditure, age, race, state, and year

^c^ Model includes adjustment for cigar use

^d^ Model includes adjustment for smokeless tobacco use

While there was no effect of cigar taxes on adolescent cigar use, cigarette taxes increased cigar use among males and females (Table 3). A 10% increase in cigarette taxes was associated with a 1.5 percentage point increase in cigar use among males (*p* = .001). Regarding frequency of use, a 10% increase in cigarette taxes was associated with males being 1.5% less likely to use cigars 0 days/month and comparatively 0.9% more likely for 1-5 days, 0.3% for 6-29 days, and 0.2% for 30 days (all *p* < .001) (Table 4). Similarly among females, a 10% increase in cigarette taxes was associated with a 0.7 percentage point increase in cigar use (*p* = .02). A 10% increase in cigarette taxes was associated with females being 0.8% less likely to use cigars 0 days/month and comparatively 0.5% more likely for 1-5 days, 0.2% for 6-29 days, and 0.1% for 30 days (all *p* = .007). However, we found no evidence for an effect of smoke-free legislation on cigar use.

As a robustness check, we only included those states that had tax changes for chewing tobacco and cigars (Additional file 1: Tables S2 and S3). The results were consistent for males and females.

## Discussion

Using representative data from 36 states over the last 15 years, we found no evidence for an effect of chewing tobacco taxes on adolescent smokeless tobacco use and similarly no evidence for an effect of cigar taxes on adolescent cigar use. In contrast, cigarette tax increases were associated with higher use and frequency of smokeless tobacco among males only, but higher use and frequency of cigars among both males and females. Among males, a 10% increase in cigarette taxes was associated with a 1.0 and 1.5 percentage point increase in the use of smokeless tobacco and cigars, respectively. Among females, a 10% increase in cigarette taxes was associated with a 0.7 percentage point increase in the use of cigars only. We also found some evidence that the implementation of smoke-free legislation increased use of smokeless tobacco among adolescent males. Our findings suggest that higher state cigarette taxes are associated with adolescents’ use of cheaper, alternative tobacco products such as smokeless tobacco and cigars.

Our study contributes to the small body of research that has examined the role of tobacco control policies on adolescent smokeless tobacco and cigar use. Using a natural experiment created through tax increases and the enactment of smoke-free legislation across and within US states, we used difference-in-differences models to test the impact of policy changes on adolescent tobacco behaviors. We found that neither chewing tobacco nor cigar taxes had an effect on tobacco use, which is in contrast to the prior studies on smokeless tobacco [11, 12] and cigars [13]. Similar to the former two studies, we estimated our models for males and our results were consistent. Considering the disproportionate use of smokeless tobacco among males [1, 6], these findings are not surprising. However, even though adolescent males were twice as likely to use cigars as females, we showed that cigarette taxes impacted cigar use among all adolescents.

While we found some evidence that the enactment of smoke-free policies may increase smokeless tobacco use among adolescent males, neither prior study included smoke-free legislation [11, 12]. Cross-sectionally, Ringel and colleagues found that adolescent cigar use was associated with cigar taxes and not smoke-free legislation [13]. Using the YRBS, we previously found that smoke-free restaurant legislation was associated with an overall reduction in adolescent smoking [10], but we did not examine alternative tobacco products. In the present study, we found no evidence that smoke-free legislation reduced cigar use. Smoke-free restaurant legislation reduces opportunities for using combustible tobacco products, but our findings suggest that adolescent males may be using smokeless tobacco as an alternative product. While there are municipalities with smoke-free policies without state-wide legislation, YRBS data are not available below the state level, suggesting that our results may underestimate the true effects of smoke-free policies on alternative tobacco products. Since 22 states had both smoke-free workplaces and restaurants, we used restaurant legislation as a proxy for state policies and it was not possible to tease apart differential effects for smoke-free workplaces on adolescent tobacco use. The current policy climate and social norms around tobacco use are very different from 2001 and earlier, when data from these studies were collected [11–13], and likely the reasons for the discrepancies. Additional studies using alternative datasets and states are needed to corroborate our findings.

One of the most effective tobacco control strategies to reduce tobacco use is taxation and adolescents are particularly price sensitive [2, 10, 22]. We have also shown that cigarette tax increases were associated with reductions in smoking for 14- and 15-year-olds, but not among older adolescents [10]. State taxes on cigarettes have increased substantially over the past decade [15] and federal cigarette taxes are higher than taxes on other tobacco products. Currently, federal taxes for cigarettes are $1.01 per pack compared to $0.0315 per 1 oz tin or pouch of chewing tobacco and 52.75% of the sales price of individual large cigars ($0.4026 maximum) [23]. Among the 34 states with percentage chewing tobacco taxes, the median (mean) percentage of sales price was 30% (43%). If chewing tobacco sells for more than $0.105 per ounce, then the state tax would be higher than the federal tax in the median state. Among the 35 states with percentage cigar taxes, the median (mean) percentage of sales price was 24% (33%). A cigar selling for more than $0.76 would face a declining federal tax rate. For lower-priced cigars, federal taxes would represent about 69% of the total tax in the median state. As we found that cigarette tax increases were associated with greater adolescent use of smokeless tobacco and cigars, adolescents are likely substituting products due to cigarettes’ higher price. In contrast, one prior study reported that higher cigarette taxes were associated with lower adolescent smokeless tobacco use [11] and the other reported no association between cigarette taxes and cigar use [13]. Among adults, Delnevo and colleagues found an increase in cigar use among recent quitters of cigarettes after New Jersey increased their cigarette tax by $0.70 per pack while the cigar tax was maintained [24]. Using repeated cross-sectional surveys from Spain, Sureda and colleagues found that younger adults had an increased prevalence of roll-your-own cigarettes over manufactured cigarettes likely in response to increases in taxation on the latter; thus, making roll-your-own cigarettes a cheaper alternative for tobacco users [25].

There has been little change in policies governing alternative tobacco products. In our data, from 1999 through 2013, 30/33 states increased their cigarette tax while 16/33 states increased their taxes on chewing tobacco or cigars (3 states either started from 0 tax or had only 1 year of data). As a percent of the retail price, this translates to a 50% average increase in cigarette taxes compared to a 73% and 58% average increase in chewing tobacco and cigar taxes, respectively. In contrast, combined with the federal tax increase in 2009, cigarette taxes ($/pack) increased by 141% in real terms over the study period. A 10% increase in cigarette taxes corresponded to a 2.9% increase in the average retail price of cigarettes, which suggests that retailers are not fully passing on tax increases to consumers. As of the second quarter in 2016, the smokeless tobacco tax in 6 states was calculated as price per ounce and the cigar tax in 5 states was calculated as price per cigar [15]. Unless taxes are inflation-adjusted, and consumers experience the full impact of tax increases, their impact diminishes over time. If taxes on alternative tobacco products are not increased in line with cigarette taxes, some of the decrease in cigarette use [1, 2] may be adolescents switching to other products, similar to what has been observed in adults [24, 25]. This would not be considered a public health success, as health risks from alternative tobacco products are the same or greater than cigarettes [2, 26, 27].

We recognize there are limitations in the YRBS. Reporting bias may underestimate the prevalence of adolescent tobacco use; however, previous research has found that self-report of smoking was a valid indicator [28], although the applicability to self-reported alternative tobacco products is not known. The YRBS is representative of students enrolled in high school only and does not collect information on household income or student employment status. Although we used rigorous econometric models to infer product substitution, unmeasured time-varying confounding could still affect the results. Since adolescents that smoked cigarettes were more likely to be excluded from our analysis due to missing data, then our effect sizes are likely underestimates of the true associations between taxes and alternative tobacco products. In the YRBS no information is captured on adolescent attitudes or reasons for smoking alternative tobacco products. Qualitative studies would provide insight into potential changes in adolescent behavior in response to policy changes.

The prevalence of adolescent smokeless tobacco and cigar use has stagnated and may, in fact, be increasing. In models adjusted for state and participant characteristics we found an increasing trend in smokeless tobacco use and no differences in cigar use over the study period. As policies on cigarettes have strengthened, marketing and sales of smokeless tobacco and cigars have increased [29–31]. The Family Smoking Prevention and Tobacco Control Act bans flavored cigarettes, except menthol, but flavors are still permissible in other products [32]. There is evidence that non-cigarette tobacco products have been reformulated to appeal to new users, including the introduction of flavors as well as changing the size of products and packaging [4, 31, 33–35].

## Conclusions

While we found no evidence that chewing tobacco or cigar taxes were effective at curbing tobacco use, increases in cigarette taxes were associated with greater use of alternative tobacco products. Taken together, this suggests that the higher price of cigarettes may be encouraging adolescents to substitute smokeless tobacco and cigars.

We also found some evidence that the enactment of smoke-free legislation increased use of smokeless tobacco among adolescent males. Reducing adolescent tobacco use will require comprehensive tobacco control policies [36] that are applied equally to and inclusive of all tobacco products.

## Additional file

Additional file 1: Table S1. Adolescent socio-demographic characteristics of the number of days/months used smokeless tobacco or cigars (*N* = 499,381). **Table S2.** Marginal effects from sex-stratified fixed-effects probit regression models of the impact of state tobacco control policies on smokeless tobacco and cigar use among those states with tax changes. **Table S3.** Marginal effects from sex-stratified fixed-effects ordered probit regression models of the impact of state tobacco control policies on the number of days/month used smokeless tobacco or cigars among those states with tax changes: Youth Risk Behavior Survey, 1999-2013. **Figure S1.** Prevalence of smokeless tobacco use by each policy: smoke-free legislation (comparing 22 states that implemented smoke-free legislation with those that did not), chewing tobacco taxes (comparing 19 states that increased chewing tobacco taxes versus those that did not), and cigarette taxes (comparing states median cigarette tax as a percentage of the price): Youth Risk Behavior Survey, 1999-2013. **Figure S2.** Prevalence of cigar use by each policy: smoke-free legislation (comparing 22 states that implemented smoke-free legislation with those that did not), cigar taxes (comparing 20 states that increased cigar taxes versus those that did not), and cigarette taxes (comparing states median cigarette tax as a percentage of the price): Youth Risk Behavior Survey, 1999-2013. (DOCX 149 kb)

## Abbreviations

CDC: Centers for disease control and prevention
YRBS: Youth risk behavior survey
