# Supplementary material for: Impact of tobacco control policies on adolescent smokeless tobacco and cigar use: a difference-in-differences approach
Source: BMC Public Health. 2018 Feb 15;18:154. doi: 10.1186/s12889-018-5063-z (PMC5813367; doi:10.1186/s12889-018-5063-z)

**Additional file 1**

**Table S1.** Adolescent socio-demographic characteristics of the number of days/months used smokeless tobacco or cigars (*N* = 499 381)

|  | Number of days/month smokeless tobacco used | | | | Number of days/month cigars used | | | |
| --- | --- | --- | --- | --- | --- | --- | --- | --- |
|  | %^a^ 0  days  (*N* =  464 132) | %^a^ 1-5 days  (*N* =  172 60) | %^a^ 6-29 days  (*N* =  9 405) | %^a^ 30 days  (*N* =  8 584) | %^a^ 0  days  (*N* =  441 355) | %^a^ 1-5 days  (*N* =  41 421) | %^a^ 6-29 days  (*N* =  11 749) | %^a^ 30 days  (*N* =  4 856) |
| Age |  |  |  |  |  |  |  |  |
| 14 | 95.7 | 2.4 | 1.1 | 0.8 | 93.4 | 4.8 | 1.3 | 0.5 |
| 15 | 94.0 | 3.3 | 1.6 | 1.2 | 90.9 | 6.7 | 1.8 | 0.7 |
| 16 | 92.7 | 3.5 | 2.0 | 1.8 | 87.8 | 8.6 | 2.7 | 0.9 |
| 17 | 92.1 | 3.5 | 2.3 | 2.1 | 85.4 | 10.6 | 2.9 | 1.2 |
| 18 | 90.3 | 3.7 | 2.8 | 3.2 | 81.5 | 12.8 | 3.8 | 2.0 |
| Race/ethnicity |  |  |  |  |  |  |  |  |
| White | 91.1 | 4.2 | 2.5 | 2.3 | 87.0 | 9.7 | 2.5 | 0.8 |
| Black | 98.0 | 1.1 | 0.4 | 0.4 | 89.8 | 6.1 | 2.6 | 1.4 |
| Hispanic | 95.2 | 2.3 | 1.2 | 1.3 | 88.9 | 7.3 | 2.4 | 1.4 |
| Other | 93.6 | 2.9 | 1.6 | 1.9 | 88.6 | 7.5 | 2.4 | 1.5 |
| Sex |  |  |  |  |  |  |  |  |
| Female | 98.0 | 1.4 | 0.3 | 0.2 | 92.3 | 5.9 | 1.4 | 0.5 |
| Male | 87.7 | 5.3 | 3.6 | 3.4 | 83.2 | 11.6 | 3.6 | 1.6 |
| Year |  |  |  |  |  |  |  |  |
| 1999 | 91.0 | 4.9 | 2.4 | 1.8 | 80.9 | 14.2 | 3.7 | 1.1 |
| 2001 | 92.8 | 3.9 | 1.8 | 1.6 | 86.4 | 10.0 | 2.7 | 0.9 |
| 2003 | 93.9 | 2.9 | 1.6 | 1.5 | 88.4 | 8.4 | 2.3 | 0.8 |
| 2005 | 93.3 | 3.2 | 1.9 | 1.6 | 87.6 | 9.1 | 2.5 | 0.9 |
| 2007 | 93.2 | 3.2 | 1.9 | 1.7 | 88.1 | 8.5 | 2.5 | 1.0 |
| 2009 | 92.5 | 3.5 | 2.1 | 1.9 | 87.5 | 8.7 | 2.7 | 1.2 |
| 2011 | 92.1 | 3.6 | 2.1 | 2.3 | 88.2 | 8.5 | 2.3 | 1.0 |
| 2013 | 93.2 | 3.0 | 2.0 | 1.8 | 89.7 | 7.0 | 2.2 | 1.1 |
| Current cigarette use |  |  |  |  |  |  |  |  |
| No | 96.4 | 1.9 | 0.9 | 0.8 | 95.1 | 3.9 | 0.8 | 0.2 |
| Yes | 77.7 | 9.5 | 6.5 | 6.2 | 56.1 | 29.5 | 10.0 | 4.4 |
| Current smokeless tobacco use |  |  |  |  |  |  |  |  |
| No | - | - | - | - | 90.6 | 7.1 | 1.8 | 0.5 |
| Yes | - | - | - | - | 50.8 | 29.8 | 12.3 | 7.1 |
| Current cigar use |  |  |  |  |  |  |  |  |
| No | 95.9 | 2.1 | 1.0 | 1.0 | - | - | - | - |
| Yes | 71.4 | 12.4 | 8.5 | 7.8 | - | - | - | - |

^a^ Weighted

**Table S2.** Marginal effects from sex-stratified fixed-effects probit regression models of the impact of state tobacco control policies on smokeless tobacco and cigar use among those states with tax changes

|  | Smokeless tobacco  (*N* = 335 985) | | | | Cigars  (*N* = 264 655) | | | |
| --- | --- | --- | --- | --- | --- | --- | --- | --- |
|  | Mean  %^a^ Use | Marginal effect of coefficient^b,c^  95% CI | | p  Value | Mean  %^a^ Use | Marginal effect of coefficient^b,d^  95% CI | | p  Value |
| Robustness check^e^ |  |  |  |  |  |  |  |  |
| Males | 10.9% |  | |  | 17.0% |  | |  |
| Tobacco tax (%) |  | -0.0001 (-0.0002-0.0001) | | .6 |  | -0.0003 (-0.0007-0.0001) | | .09 |
| Cigarette tax (%) |  | 0.0010 (0.0002-0.0018) | | .01 |  | 0.0018 (0.0006-0.0030) | | .005 |
| 100% smoke-free restaurants (yes/no) |  | 0.0098 (-0.0001-0.0194) | | .05 |  | -0.0038 (-0.0166-0.0090) | | .6 |
| Females | 1.9% |  | |  | 7.0% |  | |  |
| Tobacco tax (%) |  | -0.0000 (-0.0001-0.0000) | | .4 |  | -0.0000 (-0.0003-0.0002) | | .8 |
| Cigarette tax (%) |  | 0.0002 (-0.0002-0.0005) | | .3 |  | 0.0008 (-0.0000-0.0016) | | .06 |
| 100% smoke-free restaurants (yes/no) |  | 0.0012 (-0.0042-0.0067) | | .7 |  | 0.0054 (-0.0035-0.0142) | | .2 |

^a^ Weighted

^b^ Model includes adjustment for the following covariates: cigarette use, state tobacco control expenditure, age, race, state, and year

^c^ Model includes adjustment for cigar use

^d^ Model includes adjustment for smokeless tobacco use

^e^ Only includes states with tax changes for chewing tobacco (18 states) or cigars (16 states) from 1999-2013

CI = confidence interval

**Table S3.** Marginal effects from sex-stratified fixed-effects ordered probit regression models of the impact of state tobacco control policies on the number of days/month used smokeless tobacco or cigars among those states with tax changes: Youth Risk Behavior Survey, 1999-2013

|  | Number of days/month  smokeless tobacco used  (*N* = 335 985) | | | | Number of days/month  cigars used  (*N* = 264 655) | | | |
| --- | --- | --- | --- | --- | --- | --- | --- | --- |
|  | Mean  %^a^ Use | Marginal effect of coefficient^b,c^  95% CI | | p  Value | Mean  %^*^ Use | Marginal effect of coefficient^b,d^  95% CI | | p  Value |
| Robustness check^e^ |  |  |  |  |  |  |  |  |
| Males |  |  | |  |  |  | |  |
| 0 days | 89.1% |  | |  | 83.0% |  | |  |
| 1-5 days | 5.0% |  | |  | 12.2% |  | |  |
| 6-29 days | 3.2% |  | |  | 3.4% |  | |  |
| 30 days | 2.8% |  | |  | 1.4% |  | |  |
| Tobacco tax (%) |  |  | |  |  |  | |  |
| 0 days |  | 0.0001 (-0.0001-0.0002) | | .5 |  | 0.0003 (-0.0001-0.0006) | | .1 |
| 1-5 days |  | -0.0000 (-0.0001-0.0000) | | .5 |  | -0.0002 (-0.0004-0.0000) | | .1 |
| 6-29 days |  | -0.0000 (-0.0001-0.0000) | | .5 |  | -0.0001 (-0.0001-0.0000) | | .1 |
| 30 days |  | -0.0000 (-0.0001-0.0000) | | .5 |  | -0.0000 (-0.0001-0.0000) | | .1 |
| Cigarette tax (%) |  |  | |  |  |  | |  |
| 0 days |  | -0.0010 (-0.0018--0.0001) | | .02 |  | -0.0017 (-0.0028--0.0006) | | .003 |
| 1-5 days |  | 0.0004 (0.0001-0.0007) | | .02 |  | 0.0011 (0.0004-0.0018) | | .003 |
| 6-29 days |  | 0.0003 (0.0000-0.0005) | | .02 |  | 0.0004 (0.0001-0.0006) | | .003 |
| 30 days |  | 0.0003 (0.0000-0.0006) | | .02 |  | 0.0002 (0.0001-0.0004) | | .003 |
| 100% smoke-free restaurants (yes/no) |  |  | |  |  |  | |  |
| 0 days |  | -0.0097 (-0.0194-0.0001) | | .05 |  | 0.0004 (-0.0086-0.0159) | | .6 |
| 1-5 days |  | 0.0038 (-0.0001-0.0076) | | .05 |  | -0.0002 (-0.0103-0.0055) | | .6 |
| 6-29 days |  | 0.0027 (-0.0000-0.0055) | | .05 |  | -0.0008 (-0.0036-0.0019) | | .6 |
| 30 days |  | 0.0031 (-0.0000-0.0006) | | .05 |  | -0.0005 (-0.0021-0.0011) | | .6 |
| Females |  |  | |  |  |  | |  |
| 0 days | 98.1% |  | |  | 93.0% |  | |  |
| 1-5 days | 1.3% |  | |  | 5.4% |  | |  |
| 6-29 days | 0.3% |  | |  | 1.1% |  | |  |
| 30 days | 0.2% |  | |  | 0.4% |  | |  |
| Tobacco tax (%) |  |  | |  |  |  | |  |
| 0 days |  | 0.0001 (-0.0000-0.0002) | | .2 |  | 0.0000 (0.0003-0.0003) | | 0.9 |
| 1-5 days |  | -0.0000 (-0.0001-0.0000) | | .2 |  | -0.0000 (-0.0000-0.0010) | | 0.9 |
| 6-29 days |  | -0.0000 (-0.0000-0.0000) | | .2 |  | -0.0000 (-0.0001-0.0001) | | 0.9 |
| 30 days |  | -0.0000 (-0.0000-0.0000) | | .2 |  | -0.0000 (-0.0000-0.0000) | | 0.9 |
| Cigarette tax (%) |  |  | |  |  |  | |  |
| 0 days |  | -0.0002 (-0.0006-0.0002) | | .3 |  | -0.0007 (-0.0015-0.0000) | | .07 |
| 1-5 days |  | 0.0001 (-0.0001-0.0004) | | .3 |  | 0.0005 (-0.0000-0.0010) | | .07 |
| 6-29 days |  | 0.0000 (-0.0000-0.0001) | | .3 |  | 0.0001 (-0.0000-0.0003) | | .07 |
| 30 days |  | 0.0000 (-0.0000-0.0001) | | .3 |  | 0.0001 (-0.0000-0.0002) | | .07 |
| 100% smoke-free restaurants (yes/no) |  |  | |  |  |  | |  |
| 0 days |  | -0.0015 (-0.0067-0.0038) | | .6 |  | -0.0047 (-0.0132-0.0037) | | .3 |
| 1-5 days |  | 0.0009 (-0.0024-0.0042) | | .6 |  | 0.0031 (-0.0024-0.0086) | | .3 |
| 6-29 days |  | 0.0003 (-0.0008-0.0014) | | .6 |  | 0.0010 (-0.0008-0.0027) | | .3 |
| 30 days |  | 0.0003 (-0.0007-0.0012) | | .6 |  | 0.0007 (-0.0005-0.0019) | | .3 |

^a^ Weighted

^b^ Model includes adjustment for the following covariates: cigarette use, state tobacco control expenditure, age, race, state, and year

^c^ Model includes adjustment for cigar use

^d^ Model includes adjustment for smokeless tobacco use

^e^ Only includes states with tax changes for chewing tobacco (18 states) or cigars (16 states) from 1999-2013

CI = confidence interval

**Figure S1.** Prevalence of smokeless tobacco use by each policy: smoke-free legislation (comparing 22 states that implemented smoke-free legislation with those that did not), chewing tobacco taxes (comparing 19 states that increased chewing tobacco taxes versus those that did not), and cigarette taxes (comparing states median cigarette tax as a percentage of the price): Youth Risk Behavior Survey, 1999-2013


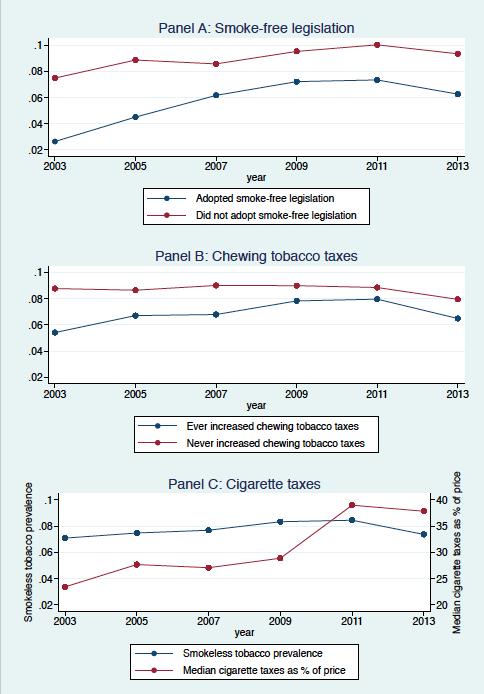


**Figure S2.** Prevalence of cigar use by each policy: smoke-free legislation (comparing 22 states that implemented smoke-free legislation with those that did not), cigar taxes (comparing 20 states that increased cigar taxes versus those that did not), and cigarette taxes (comparing states median cigarette tax as a percentage of the price): Youth Risk Behavior Survey, 1999-2013


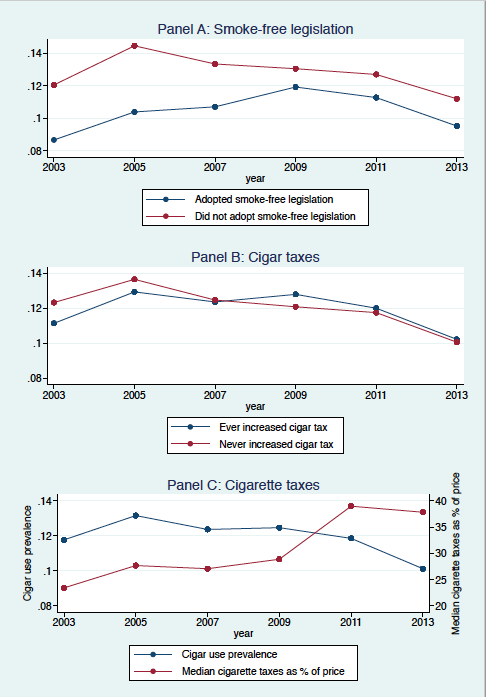

Supplement: Supplementary file 1 — Adolescent socio-demographic characteristics of the number of days/months used smokeless tobacco or cigars (N = 499,381). Table S2. Marginal effects from sex-stratified fixed-effects probit regression models of the impact of state tobacco control policies on smokeless tobacco and cigar use among those states with tax changes. Table S3. Marginal effects from sex-stratified fixed-effects ordered probit regression models of the impact of state tobacco control policies on the number of days/month used smokeless tobacco or cigars among those states with tax changes: Youth Risk Behavior Survey, 1999-2013. Figure S1. Prevalence of smokeless tobacco use by each policy: smoke-free legislation (comparing 22 states that implemented smoke-free legislation with those that did not), chewing tobacco taxes (comparing 19 states that increased chewing tobacco taxes versus those that did not), and cigarette taxes (comparing states median cigarette tax as a percentage of the price): Youth Risk Behavior Survey, 1999-2013. Figure S2. Prevalence of cigar use by each policy: smoke-free legislation (comparing 22 states that implemented smoke-free legislation with those that did not), cigar taxes (comparing 20 states that increased cigar taxes versus those that did not), and cigarette taxes (comparing states median cigarette tax as a percentage of the price): Youth Risk Behavior Survey, 1999-2013. (DOCX 149 kb) [file 12889_2018_5063_MOESM1_ESM.docx]
